# Enhanced Cadmium Detection in Real Water Sources Using a Custom Nanocomposite-Modified Carbon Electrode and Optimized Square Wave Voltammetry

**DOI:** 10.3390/s26196112

**Published:** 2026-09-26

**Authors:** Dipen Basnet, Rohit Boddu, Xiyuan Liu, Prabhu U. Arumugam, Shabnam Siddiqui

**Affiliations:** 1 Institute for Micromanufacturing (IfM), Louisiana Tech University, Ruston, LA 71272, USAparumug@office.latech.edu (P.U.A.); 2 Department of Mathematics and Statistics, Louisiana Tech University, Ruston, LA 71272, USA; liuxyuan@latech.edu; 3 Center for Biomedical Engineering and Rehabilitation Science (CBERS), Louisiana Tech University, Ruston, LA 71272, USA

**Keywords:** electrochemical sensors, nanocomposite, heavy metals, cadmium, square wave voltammetry, fast causal inference, carbon, water monitoring

## Abstract

Monitoring toxic heavy metals in complex, real water sources is essential for safe water supplies. Current sensors lack the sensitivity, selectivity, and limits of detection (LOD) needed to detect heavy metals such as cadmium (Cd^2+^) in real water samples with minimal preparation. Here, we demonstrate a novel ternary nanocomposite-coated carbon electrochemical sensor that detects Cd^2+^ with the highest sensitivity (14.71 ± 0.3, 6.94 ± 0.8 μA·ppb^−1^·cm^−2^) and the lowest LODs (0.06 ± 0.02, 0.61 ± 0.10 ppb) in spiked deionized and lake waters, respectively. Sensor fabrication optimization included electrochemical polishing of carbon screen-printed electrodes, surface modification with a 1 mg/mL bismuth-reduced graphene–molybdenum disulfide nanocomposite ink, and rapid heating and cooling at 75 °C for 3 min. These steps, along with square wave anodic stripping voltammetry (SWASV) deposition potential identification, produced distinct detection peaks and high reproducibility (8% RSD). We further investigated the SWASV parameters, specifically frequency (5 to 50 Hz), amplitude (1 to 100 mV), and step potential (5 to 70 mV), using a fast causal inference algorithm, which resulted in a 233-fold increase in Cd^2+^ sensitivity. We found that the LOD and the full width at half maximum of the Cd^2+^ peak are dependent on frequency, while sensitivity and peak resolution depend on amplitude and step potential. Under optimal detection conditions of 35 Hz, 60 mV amplitude, and a 10 mV step potential, the sensor showed adequate sensitivity and LODs, even in the presence of 10-fold concentrations of Zn^2+^, As^3+^, and Cu^2+^ as interferents, confirming its suitability for real-world water-quality field testing.

## 1. Introduction

Toxic heavy metal contamination in drinking, well, and surface waters has become a significant threat to environmental and public health, necessitating advanced electroanalytical techniques that are capable of reliably detecting them at ultra-trace levels (below parts per billion, ppb) [1]. Among the many heavy metals, cadmium (Cd^2+^) has a greater tendency towards bioaccumulation, high toxicity, and a long biological half-life [2]. Regulatory agencies such as the World Health Organization (WHO) and the United States Environmental Protection Agency (EPA) have established MCLs of 3 µg/L (3 ppb) and 5 µg/L (5 ppb), respectively, for Cd^2+^ in drinking and well waters. This poses demanding requirements on the Cd^2+^ sensors that are expected to demonstrate exceptional analytical sensitivity, reliability, and reproducibility [1]. Traditional laboratory-based techniques, such as inductively coupled plasma mass spectroscopy (ICP-MS) and atomic absorption spectroscopy (AAS), have provided excellent sensitivity and limit of detection (LOD) ng/L (ppt) to low ppb ranges but are poorly suited for large-scale, on-site, on-demand, real-time monitoring due to high cost, complex instrumentation, multi-step sample preparation requiring expensive reagents, and reliance on qualified technicians [3,4,5]. These limitations have motivated researchers to develop next-generation sensors that are portable, rapid, sensitive, selective, and can detect Cd^2+^ at sub-ppb levels in real water sources with minimal to no sample preparation.

Among sensing modalities, electrochemical methods are an efficient option for trace-level Cd^2+^ detection because of their intrinsic preconcentration capabilities, high selectivity (Cd^2+^ has its own potential window where it is anodically stripped, −0.9 to −0.7 V), and compatibility with miniature electrodes [6], especially square wave anodic stripping voltammetry (SWASV), which provides significant advantages for Cd^2+^ detection that include high sensitivity, quick analysis times, and effective suppression of capacitive background currents and non-faradaic contributions [7,8,9,10]. Among electrochemical sensor materials, carbon is strongly preferred as the base electrode material due to its rich surface chemistry, high electrical conductivity, and exceptional electrochemical properties [8,9]. Electrode pretreatments, such as electrochemical polishing (ECP), electrochemical roughening, laser etching, and plasma etching, are commonly used to activate the carbon electrode surface, thereby boosting sensor metrics such as sensitivity, signal-to-noise ratio, and LODs. Also, they are generally surface-modified with a variety of electrocatalysts, including bismuth (Bi), molybdenum disulfide (MoS_2_), reduced graphene oxide (rGO) [10,11,12,13,14], gold nanoparticles [15,16] (AuNPs), carbon nanotubes (CNTs) [17], graphene [18], metal–organic frameworks (MOFs) [19], and conducting polymers [20] to achieve the necessary sensitivity, selectivity, and LOD required for real-world sensing applications. Nanostructured and nanocomposite-modified electrodes have been widely explored for sensitive electrochemical detection of toxic metal ions in complex aqueous matrices because they can enhance electroactive surface area, ion adsorption, and interfacial electron transfer. Such composite interfaces have shown improved analytical performance for heavy metal detection in water and other complex samples [21]. Bi, a green alternative to the widely used mercury electrode, forms stable alloys with heavy metals, including Cd^2+^ and Pb, similar to those formed by mercury amalgam. Moreover, Bi-modified electrodes exhibit relatively high sensitivity and a wide working potential range that is hardly affected by dissolved oxygen, helping reduce Cd^2+^ to form a Cd-Bi alloy during the SWASV preconcentration step and producing well-defined detection peaks [22,23]. rGO provides a conductive, percolated electron-transport network that enhances charge transfer while offering surface functional groups, restoring much of the π-conjugated carbon network and making it an excellent electron transporter that supports Cd^2+^ adsorption [24]. MoS_2_, a layered, hybrid transition-metal dichalcogenide, enables the formation of novel heterojunctions, promotes strong adsorption (via sulfur bonds) with additional catalytic sites, and exhibits strong-ion affinity, arising from its edge-rich structure and fast electron transfer [25,26].

Previous studies have demonstrated the individual advantages of Bi-, graphene-, and MoS_2_-based materials for electrochemical heavy metal sensing. Bismuth-based electrodes are well established in anodic stripping analysis of Cd^2+^ because of their good stripping characteristics, large cathodic potential range, and as an environmentally friendly alternative to mercury-based electrodes [27]. Reduced graphene oxide has also been applied to highly sensitive Cd^2+^ detection and has been shown to serve as an electrically conductive, carbon-based platform for electrochemical signal transduction [28]. In contrast, MoS_2_ has a large surface area and more sulfur-containing adsorption sites with affinity for Cd^2+^, facilitating metal-ion accumulation at the sensing interface [29]. In addition, previous research has shown that coupling graphene and MoS_2_ yield significantly superior Cd^2+^ responses, suggesting that graphene’s charge-transport properties and MoS_2_’s adsorption properties can create a beneficial interfacial effect in the composite. For instance, Feng et al. fabricated a CuO nanoparticle-based electrochemical sensing interface and demonstrated higher electron-transfer kinetics and catalytic activity, showing the versatility of the nanomaterial-enhanced electrode interface for high-sensitivity electrochemical detection [30].

In this work, we investigated a new ternary Bi_2_O_2_CO_3_-rGO-MoS_2_ nanocomposite material, synthesized, formulated, and first tested for Cd^2+^ sensing in spiked DI water and then in lake water samples, demonstrating its practical utility in real-world scenarios. Particularly, tuning the nanocomposite composition, loading, and geometry (size/shape), aided by an indirect nano-sonication process before its application onto a carbon screen-printed electrode (cSPE), positively affected the surface area, number of active sites, electrode–electrolyte interface, Cd^2+^ preconcentration, alloy formation, and detection peak currents [20,22,23]. We hypothesize that the ternary interface synergizes the complementary properties of Bi-assisted Cd^2+^ preconcentration/stripping, charge transport through rGO, and adsorption sites from MoS_2_ in a single sensing layer, yielding the highest currents and sensitivities towards Cd^2+,^, and our experimental data demonstrate that this is true, in comparison to either single or their respective binary combinations. Secondly, we carried out a systematic study of the effect of the SWASV parameters (frequency, amplitude, and step potential) on the key sensor performance metrics (sensitivity, LOD, full width at half maximum, FWHM) using a carefully designed set of 76 experiments and applying fast causal inference (FCI) modeling for the data analysis, as the analytical performance of SWASV is strongly governed by the selection of these three waveform parameters [7,10,31].

## 2. Materials and Methods

### 2.1. Chemicals

The chemicals purchased from Sigma Aldrich (St. Louis, MO, USA) for this work included sulfuric acid (CAS No. 7664-93-9), potassium chloride (CAS No. 7447-40-7), potassium hexacyanoferrate (II) trihydrate (CAS. 14459-95-1), potassium hexacyanoferrate (III) (CAS No. 13746-66-2), bismuth (III) nitrate pentahydrate (CAS No. 10035-06-0), graphene oxide powder, molybdenum disulfide (CAS No. 1317-33-5), ethylene glycol (CAS No. 107-21-1), sodium borohydride (CAS No. 16940-66-2), dimethylformamide (CAS No. 68-12-2), sodium acetate (CAS No. 127-09-3), acetic acid (CAS No. 64-19-7), cadmium standard for AAS (Product No. 51994), and lead standard for AAS (Product No. 16595). We used a saturated calomel reference electrode (RE) (Gamry Instruments, Warminster, PA, USA) for the ECP pretreatment.

### 2.2. Synthesis of Bi_2_O_2_CO_3_-rGO-MoS_2_ Ternary Nanocomposite

We synthesized the Bi_2_O_2_CO_3_-rGO-MoS_2_ ternary nanocomposite via chemical reduction using a mixed-solvent system. We prepared a total of 20 mL of solvent by combining 10 mL of deionized (DI) water with 10 mL of ethylene glycol. To this, 100 mg each of bismuth nitrate pentahydrate (Bi (NO_3_)_3_·5H_2_O), graphene oxide (GO), and MoS_2_ was added. The suspension was magnetically stirred at 300 rpm and 60 °C for 30 min to ensure a homogeneous dispersion. After dispersion, chemical reduction was initiated by gradually adding 0.51 g of sodium borohydride in portions under continuous stirring at 60 °C. The reduction process was maintained for 2 h to ensure effective reduction in GO and incorporation of Bi nanoparticles into the MoS_2_ and rGO matrix. After the reaction, the suspension was centrifuged at 4500 rpm for 10 min to collect the nanocomposite precipitate. The centrifugation and washing process was repeated three times with DI water to remove unreacted precursors and solvent residues. The final nanocomposite product was dried in a glass Petri dish at 50 °C overnight. For cSPE modification, 1 mg of dried nanocomposite was dispersed in 1 mL of dimethylformamide and sonicated for 20 min to obtain a stable, homogeneous suspension suitable for drop-casting onto the commercially purchased eight working cSPEs patterned in a circular fashion (Figure 1a) (see Supplementary Information, Figure S1).

### 2.3. Surface, Electrical, and Raman Measurements

The surface morphology and nanocomposite composition were characterized using a field-emission scanning electron microscope (FESEM: Hitachi S-4800, Schaumburg, IL, USA). We obtained X-ray Photoelectron Spectroscopy (XPS) data using a ThermoFisher, Waltham, MA, USA K-Alpha XPS system with a monochromated Al X-ray source. The analysis chamber base pressure was approximately 5 × 10^−9^ mbar. We ran high-resolution scans of individual photoelectron lines at a pass energy of 20.000 eV, a step size of 0.1 eV, and a dwell time of 100 microseconds. We used a four-point probe system (Jandel RM3000, Leighton Buzzard, Bedfordshire, UK) to assess the electrical properties. At room temperature, we measured sheet resistance by applying a constant probe current of 1 mA through the outer probes and measuring the resulting voltage drop across the inner probes. We used multiple measurements to evaluate spatial uniformity and measurement repeatability, taking four independent measurements at various locations on the electrode surface, including the center and peripheral regions. In each measurement, the four probes were in full contact with the cSPE surface. We evaluated the cSPEs for electrical conductivity and uniformity before and after nanocomposite modification using the resulting sheet resistance values. We recorded Raman spectra using a Horiba Jobin Yvon LabRam, Piscataway, NJ, USA equipped with a 40 mW He-Ne laser at an excitation wavelength of 514 nm, applied to the electrode surface.

### 2.4. Electrochemical Measurements

A STAT-i-MULTI8 multichannel potentiostat (Metrohm Dropsens µ8000P, Riverview, FL, USA) was used for all electrochemical experiments. We used a commercial chip (Metrohm, 8W110, Riverview, FL, USA) that comprises an array of eight electrically individually addressable cSPEs as working electrodes, each measuring 2.95 mm in diameter, a built-in carbon ring counter electrode, and an Ag/AgCl reference electrode located at the center (Figure 1a). The ECP was performed in 0.1 M H_2_SO_4_ by cycling the potential within a ±2 V electrochemical potential window at a fixed scan rate of 40 mV/s for 10 cycles, following a previously established protocol [11]. Cyclic voltammetry (CV) was performed at 100 mV/s using a 5 mM ferro/ferricyanide redox couple in a supporting electrolyte of 1 M KCL. Using the same solution, we recorded electrochemical impedance spectroscopy (EIS) from 0.1 Hz to 100 kHz with a 10 mV AC signal amplitude (rms) at open-circuit potential (OCP). For Cd^2+^ calibration studies, we employed a deposition potential of −1.1 V and a deposition time of 200 s. We stirred the 50 mL solution at 200 rpm using a magnetic stirrer (VWR 600 series). We varied the Cd^2+^ concentration from 0 ppb to 30 ppb. We repeated all experiments at least 3 times using three different cSPEs across multiple chips. The cyclic voltammograms, impedance spectra, and Cd^2+^ calibration plots shown below are representative of these data. We prepared all solutions on the day of the experiments and purged them with nitrogen gas for 5 min before use.

### 2.5. Buffer Preparation and Lake Water Sampling

For Cd^2+^ sensor calibration studies, we prepared a sodium acetate buffer (pH 4.7) using DI water. We received real water samples collected from East Lake, Birmingham, AL, USA, from the Wesley Zech research group at the University of Alabama at Birmingham (UAB), Birmingham, USA. We collected the water samples by inserting the sampling bottle opening just beneath the water surface, without disturbing the surface or sediments, into clean 500 mL bottles. We collected samples and immediately measured environmental parameters, including pH. We preserved one set of samples in nitric acid to keep heavy metal constituents stable, while leaving the remaining samples unpreserved to maintain their natural water chemistry for Cd^2+^ sensor evaluation. We then placed the samples in coolers with ice (around 4 °C), transported them to the UAB laboratory, and sent them to Louisiana Tech University within 48 h, following documented chain-of-custody procedures. Upon arrival, we refrigerated unpreserved samples, then used them to calibrate and recover the Cd^2+^ sensors and evaluate performance in real water. To remove suspended particulates, the lake water was filtered through filters with pore sizes of 0.45 µm or 0.2 µm (10040-464/462, VWR, Radnor, PA, USA) before being adjusted to the optimal pH of 4.7 using acetic acid and sodium acetate [11] as the lake water’s initial pH was 8.5.

## 3. Experimental Results and Analysis

### 3.1. Surface, Raman, and Electrical Characteristics of the Nanocomposite-Modified cSPEs

The SEM images show the surface morphology of the ECP-treated cSPE (Figure 1b,c) and the nanocomposite-modified, ECP-treated cSPE (Figure 1d,e). The cSPE exhibits a typical rough surface morphology with randomly distributed carbon agglomerates and valleys, resulting in a highly heterogeneous topography, as expected for a screen-printed electrode. After surface modification with the nanocomposite coating, the nanocomposite features a 2D sheet-like geometry with multiple layers of rGO and MoS_2_, decorated with microscale Bi particulates. SEM image analysis shows an average sheet size of 160 nm and a Bi particle size of 78 nm. Using ImageJ (Version: 1.54g), we randomly measured approximately 250 particles from multiple regions of the modified electrode to obtain a statistically reliable estimate of the particle-size distribution. SEM micrographs of the nanocomposite-modified cSPE (see Supplementary Information, Figure S2) demonstrate the characteristic morphology and uniform distribution of the deposited nanocomposite. Based on EDS elemental mapping and analysis (Figure 1f–h), the nanocomposite appears uniformly and randomly distributed over the cSPE, indicating successful modification. EDS confirms the presence of Bi and Mo, along with C and O (and S associated with MoS_2_), thereby verifying the successful incorporation of the Bi and MoS_2_-based components onto the cSPE. It also shows that Bi is uniformly distributed throughout the coated region, suggesting that the Bi-containing phase is not confined to isolated islands but is dispersed throughout the film. The MoS_2_ map is like Bi, indicating co-localization of MoS_2_ within the Bi-rGO binary phase rather than phase separation into distinct, non-overlapping domains.

Additionally, XPS (see Supplementary Information, Figure S3) confirmed the successful integration of Bi, rGO, and MoS_2_ in the synthesized ternary composite. The Bi 4f spectrum was dominated by Bi^3+^ peaks at approximately 159.6 and 164.8 eV, indicating oxidized Bi–O species, together with a smaller reduced Bi contribution. The C 1s peaks confirmed the presence of *sp*^2^ carbon and oxygen-containing functional groups associated with rGO, while the O 1s spectrum showed metal–oxygen and carbonate/oxygen-containing species. The Mo 3d peaks at 229.5 and 232.6 eV confirmed Mo^4+^ in MoS_2_, whereas the higher binding energy component at 235.5 eV indicated partial surface oxidation of Mo. Overall, the XPS results support the formation of a bismuth oxycarbonate (Bi_2_O_2_CO_3_)-rGO-MoS_2_ ternary composite, rather than a predominantly metallic Bi-containing composite, and confirm the coexistence of all three functional components within the nanocomposite.

Figure 1i shows the Raman spectra of the as-received cSPE, ECP-treated cSPE, and nanocomposite-modified, ECP-treated cSPE. All spectra show the typical D band at 1350 cm^−1^ and the G band at 1580 cm^−1^, characteristic of *sp*^2^-hybridized carbon materials. The D band represents structural (lattice) defects, whereas the G band represents the crystalline graphitic core and corresponds to the in-plane vibration of *sp*^2^ carbon atoms. Moreover, a weak, diffuse 2D band at approximately 2700 cm^−1^ indicates graphitic carbon with a few-layer graphene structure. The quantitative measure of structural disorder and defect density in the base cSPE was obtained from the ratio of the D- and G-band peaks/intensities (I_D_/I_G_). The as-received cSPE has a baseline ratio of 0.59, indicating intrinsic defects and active edge sites (planes) attributable to the carbon ink composition and formulation and the screen-printing coating process. After ECP, the I_D_/I_G_ ratio increased to 0.75, indicating greater structural disorder, higher defect density, and altered surface functional groups, which contribute to more electrochemically active sites and are desirable features for enhanced electrode (sensor) activity (as confirmed by electrochemical characterization studies detailed in the next section). After nanocomposite modification, the I_D_/I_G_ ratio decreases to 0.54, indicating partial restoration of the *sp*^2^ network and passivation of some defective sites and disorder introduced during ECP pretreatment, arising from the presence of conductive rGO within the nanocomposite-dispersed phase. Overall, the Raman results confirm that nanocomposite-modified, ECP-treated cSPEs retain the necessary surface disorder and electroactive sites for enhanced electrical conductivity, electroactive area, activity, and electrode kinetics.

Next, we measured the sheet resistance of the as-received, ECP-treated, and nanocomposite-modified, ECP-treated cSPEs. The as-received cSPE had an average value of 106.52 ± 1.08 Ω/□ (n = 3). After ECP pretreatment, it decreased significantly to 53.51 ± 0.86 Ω/□ (n = 3), as expected, due to the introduction of additional electroactive sites and an increased electroactive area, as evidenced by the CV and EIS measurements (details in the next section). After nanocomposite modification, the average sheet resistance slightly decreased to 51.55 ± 0.62 Ω/□ (n = 3), due to the binding of the highly conductive nanocomposite-dispersed phase, which increased lateral charge transport and created larger, more continuous conductive paths through the near-surface carbon electrode structure. We converted the sheet resistance values to bulk resistivity by assuming a homogeneous effective film thickness of 10 µm, with ρ = R_s_ × t. The resistivities of the as-received, ECP-treated, and nanocomposite-modified, ECP-treated cSPEs were ~1.07 × 10^−3^ Ω·m, 5.35 × 10^−4^ Ω·m, and 5.16 × 10^−4^ Ω·m, respectively (n = 6). The corresponding electrical conductivities (σ = 1/ρ) were 9.39 × 10^2^ S/m, 1.87 × 10^3^ S/m, and 1.94 × 10^3^ S/m, respectively, representing a 2-fold increase after the ECP pretreatment and nanocomposite modification.

### 3.2. Electrochemical Characteristics of the Nanocomposite-Modified, ECP-Treated cSPEs

The cyclic voltammograms and Nyquist plots of the as-received, ECP-treated, and nanocomposite-modified, ECP-treated cSPEs are shown in Figure 2a,b. The anodic peak currents (i*_pf_*) that represent the electroactive area of the cSPE were 84.0 ± 0.5 µA, 108.8 ± 0.2 µA, and 97.32 ± 0.8 µA (n = 3), respectively. This is likely because ECP removes surface contaminants, increases the electroactive area, and activates electroactive sites, thereby facilitating faster electron transfer at the electrode–electrolyte interface. The nanocomposite-modified cSPE exhibited a slight decrease in the electroactive area due to the ~10.5 ± 0.5% surface coverage of the nanocomposite-dispersed phase (see Supplementary Information, Figure S2) within the continuous cSPE matrix phase. This was also evident from the corresponding decrease in the I_D_/I_G_ ratio and the electrical conductivity. Secondly, the peak potential separations (E_p_) between the forward and reverse peak currents (E*_anodic_* − E*_cathodic_*) of the redox system (ΔE_p_) are a reliable CV indicator to study electrode reaction rates [32] and were 178.5 ± 2.04 mV, 123.6 ± 0.85 mV, and 119.0 ± 0.7 mV, respectively (n = 3). The ΔE_p_ value is the highest for the as-received cSPE, which exhibits the slowest electron-transfer kinetics. The lowest ΔE_p_ value of 119 mV for the nanocomposite-modified cSPE is still greater than that of a reversible two-electron redox process (29.5 mV), yet it exhibits the best electrochemical reversibility, with the lowest ΔE_p_. This is due to the synergistic effects of the electrically conductive rGO network and the electrocatalytic activity of Bi and MoS_2,_ which further improve interfacial charge-transfer properties. Faster, facile electrode kinetics with adequate electroactive areas, as demonstrated here, are crucial for achieving higher sensitivity in Cd^2+^ detection.

To further investigate the interfacial electrochemical properties of the nanocomposite-modified cSPEs, we performed EIS studies. We fitted the EIS data from the Nyquist plots (Figure 2b) to a Randles circuit (see Supplementary Information, Figure S4). The circuit suggests that the impedance of the as-received cPSE arises from the constant phase element (CPE), charge-transfer resistance (*R_ct_*), and a Warburg (diffusion) element (W). The current follows two parallel paths: one through the CPE and the other through *R_ct_* and W. After the ECP pretreatment, *R_ct_* drops significantly by a factor of 6, from 648 ± 24 Ω to 109 ± 12 Ω (n = 3), suggesting a substantial increase in the electroactive area, as confirmed by the increase in the CV peak currents and I_D_/I_G_ ratio. The diffusional impedance, which is due to the Warburg element, also decreased by ~5%. Also, the cSPE surface becomes smoother, as indicated by the fitting of a capacitive element (C) instead of a CPE (see Supplementary Information, Figure S4). This implies that the double-layer capacitance exhibits a uniform current-density distribution over the electrode surface, unlike that of as-received cSPE. Thus, ECP pretreatment significantly increased cSPE electrical conductivity. The circuit remained unchanged after nanocomposite modification, suggesting that the electrode’s overall electrochemical behavior did not change. The data suggest that the solution resistance (R_s_) of the nanocomposite-modified, ECP-treated cSPEs increased by ~76%, as evidenced by a shift in the baseline of the Nyquist plots (Figure 2b), owing to the high electroactivity and rich surface chemistry of cSPEs. The *R_ct_* increases marginally by ~12%, and the Warburg impedance increases by ~11%. However, the double-layer capacitance impedance decreases by ~56%, which suggests that the double layer has become conductive. The capacitance of a double layer is defined as C = εA/d, where A is the area of the electrode, d is the distance between the electrode and the charges on its surface, and ε is the permittivity of the medium. This equation suggests that either the double-layer area or the medium permittivity has increased due to the nanocomposite coating. Overall, the EIS results support the conclusion that ternary nanocomposite-modified, ECP-treated cSPEs have the interfacial electrochemical properties required to deliver an electrochemical sensor with superior metrics.

### 3.3. Optimization of SWASV Deposition Potential and Nanocomposite Loading

To enhance Cd^2+^ detection sensitivity further, we investigated the effects of SWASV deposition potential (Figure 3a) and the ternary nanocomposite loading (mg/mL) (Figure 3b) at a fixed Cd^2+^ concentration of 30 ppb, deposition time of 200 s, and the optimal SWASV parameters of 35 Hz frequency, 10 mV step potential, and 60 mV amplitude (details in the next section). The highest Cd^2+^ detection current was observed at −1.1 V (~30 μA). At higher cathodic potentials, undesirable reactions, such as hydrogen evolution, increased background noise [13] and affected signal intensity and quality. The detection current increased sharply from ~14 μA to 30 μA (a 114% increase) when the loading was increased from 0.5 mg/mL to 1.0 mg/mL. Further increasing the loading sharply reduced the currents to as low as ~11 μA at 4 mg/mL. This is due to a further increase in *R_ct_* from increased surface capacitance and surface charge, and a decrease in electroactive area at higher loading, as the nanocomposite-dispersed phase generally decreased the electroactive area, increased *R_ct_*, and increased capacitance. For all subsequent studies, we used −1.1 V as the SWASV deposition potential and a nanocomposite loading of 1.0 mg/mL.

### 3.4. Effect of the Nanocomposite Composition on Cd^2+^ Sensitivity

We performed a comparative study of the effect of the three principal components of the nanocomposite, Bi, MoS_2,_ and rGO, on Cd^2+^ detection for the first time. ECP-treated cSPEs were modified with single-component (Bi_2_O_2_CO_3_, MoS_2,_ and rGO), two-component, binary (Bi_2_O_2_CO_3_-rGO, Bi_2_O_2_CO_3_-MoS_2,_ and rGO-MoS_2_), and ternary (Bi_2_O_2_CO_3_-rGO-MoS_2_) nanocomposites (Figure 4). At the single component level, Bi_2_O_2_CO_3_ exhibited the maximum Cd^2+^ sensitivity of 7.65 ± 0.43 µA·ppb^−1^·cm^−2^, MoS_2_ and rGO, providing 2.37 ± 0.50 µA·ppb^−1^·cm^−2^ and 1.06 ± 0.52 µA·ppb^−1^·cm^−2^ (n = 3). At the two-component level, we observed that synergistic effects depend strongly on component pairing. The Bi_2_O_2_CO_3_-rGO pairing delivered the highest sensitivity of 9.36 ± 0.42 µA·ppb^−1^·cm^−2^, potentially because the highly electrically conductive rGO electron-transport network not only improves the interfacial electrical conductivity by reducing the *R_ct_* but also increases the availability of electrocatalytic Bi_2_O_2_CO_3_ sites. The Bi_2_O_2_CO_3_-MoS_2_ pairing demonstrated a sensitivity of 8.18 ± 0.55 µA·ppb^−1^·cm^−2^, suggesting that MoS_2_ exhibits an exceptional 2D-layered edge effect, contributing to abundant adsorption/electrocatalytic sites [14,33] and complementing Bi, which is the principal electrocatalyst and alloy-forming element for heavy metals, next to mercury [34,35]. In contrast, the rGO-MoS_2_ pairing exhibited the lowest sensitivity of 2.94 ± 0.63 µA·ppb^−1^·cm^−2^ (n = 3), suggesting a highly unfavorable synergistic effect between rGO and MoS_2_.

At the ternary level (Figure 4g), we achieved the highest Cd^2+^ sensitivity of 14.71 ± 0.3 µA·ppb^−1^·cm^−2^ (n = 3), providing strong experimental evidence for the combined effect of all three components investigated in this study. This represents a ~57% enhancement over the top-performing binary Bi_2_O_2_CO_3_-rGO electrode and a ~92% improvement over Bi alone. Analysis of single-, binary-, and ternary-component electrodes under uniform electrochemical conditions provides strong experimental evidence of a synergistic improvement in analytical performance when all three components are integrated. This improved response aligns with the synergistic roles of Bi in preconcentrating Cd^2+^ and alloy synthesis, rGO in promoting electron transfer, and MoS_2_ in providing supplementary adsorption and electrocatalytic sites. Significantly, this increased sensitivity was observed at the optimal nanocomposite concentration of 1 mg/. In contrast, higher nanocomposite loadings reduced the Cd^2+^ response, suggesting that enhanced sensor performance stems from a carefully tuned ternary interface rather than solely from increased material loading.

### 3.5. Effect of SWASV Parameters on Cd^2+^ Sensitivity

Next, we investigated three SWASV parameters: pulse frequency (Hz), pulse height (step potential, E_s_), and pulse amplitude (E_p_). Based on SWASV theory, optimal analytical performance requires that the staircase potential shift (E_s_) at the end of the step be much smaller than E_p_. Also, according to the Cottrell current equation [25], higher frequency leads to a higher plateau current, the Cottrell current for pulse width, t_p_. Based on these criteria, we systematically varied the three SWASV control parameters (variables) (see Supplementary Information, Table S1) and assessed their effects on Cd^2+^ sensitivity and LOD. The dataset includes measurements from three sensors, each with 19 replicates, for a total of 57 observations. The dataset includes measurements from three sensors, each with 19 replicates, for a total of 76 observations. The dataset features four distinct frequency levels (5, 20, 35, and 50 Hz), four amplitude levels (1, 20, 60, and 100 mV), and seven step potential levels (5, 10, 20, 30, 40, 50, and 70 mV). At each frequency, 19 selected combinations of amplitude and step potential were tested, resulting in a total of 76 experimental conditions. The observed variables include the LOD, full width at half maximum (FWHM) (at 10 ppb), and sensitivity. Figure 5 shows representative Cd^2+^ calibration plots.

The primary aim here is to investigate the correlations between these control variables and the observed variables and to identify conditions that maximize Cd^2+^ sensitivity and minimize LOD. To do so, we used the fast causal inference (FCI) algorithm to identify the relationships among the variables, followed by an analysis of the trends in each relationship. As an extension of the PC algorithm, FCI identifies conditional dependencies between variables and accounts for potential latent confounders by relaxing the causal sufficiency assumption [36]. Given the limited number of replicates (19) for each electrode in this dataset, we used the FCI algorithm, together with Fisher’s Z conditional independence test, to explore these relationships.

Figure 6a presents the correlation results between the control and observed variables, as identified by the FCI algorithm, with a Type I error rate of 0.05 for each independence test. The results show that frequency is correlated with both the LOD and the FWHM while remaining independent of sensitivity. This implies that frequency adjustments can optimize both the LOD and the FWHM. In contrast, amplitude is correlated only with sensitivity. Additionally, step potential appears to be independent of all observed variables. Sensors (1), (2), and (3) in the figure, as well as the bidirectional edges between these sensors, reflect repeated measurements. Although the FCI algorithm identified these relationships, they are less relevant to this study’s primary objective. Next, to reduce the risk of failing to detect a correlation between two variables (i.e., a Type II error), we set the significance level (i.e., Type I error) to 0.10. In this case, the conclusions change when we include step potential in the correlation. At this significance level, the conclusion changes when we include step potential in the correlation. Specifically, both step potential and frequency are associated with FWHM, compared with only frequency in Figure 6a, while the rest of the conclusions remain the same.

Hence, based on the differences observed between Figure 6a,b, we suggest that step potential has a weaker effect on FWHM, a metric of Cd^2+^ peak sharpness and resolution, than frequency. Therefore, the results indicate that frequency remains the primary driver of the FWHM. To further analyze how the frequency, amplitude, and step potential affect the observed variables, we constructed more detailed data summaries.

#### 3.5.1. Effect of SWASV Frequency on Cd^2+^ LOD

We begin by analyzing the relationship between frequency and the LOD, a key sensor performance metric. In this study, a lower LOD and lower variance indicate a more desirable outcome. Figure 7 (top row) illustrates the relationship between frequency and the LOD for three different sensors. The boxplot reveals a convex trend, with the mean LOD reaching its lowest point between 20 Hz and 35 Hz. Similarly, the variance follows a convex pattern, indicating greater stability within this frequency range. These findings suggest that setting the frequency within this range minimizes the LOD while ensuring more consistent results across sensors. Table S1 (see Supplementary Information) summarizes the data and confirms that the mean and variance for all three sensors reach their minima at 35 Hz. Among the sensors, Sensor 2 performs best at 35 Hz, with a mean LOD of 0.55 ppb and a variance of 0.18.

#### 3.5.2. Effect of SWASV Amplitude on Cd^2+^ Sensitivity

While higher amplitude enhances current response (sensitivity), it may also increase signal noise and variability. In this section, we analyze the relationship between amplitude and sensitivity, a key sensor performance metric. In this study, higher sensitivity, coupled with lower variance, is more favorable. Figure 7 (bottom row) illustrates the relationship between amplitude and sensitivity for three different sensors. The boxplot shows an increasing trend for both the mean and variance, indicating that as amplitude increases, sensitivity also increases. However, this improved sensitivity comes with increased instability, as shown by the rising variance. Table S2 (see Supplementary Information) shows the mean and variance of sensitivity for each sensor at different amplitude levels, while Figure S5 (see Supplementary Information) visualizes these data. The optimal point in the figure is in the bottom-right corner, corresponding to the largest mean and smallest variance. Based on this observation, Sensor 1 achieves optimal sensitivity at an amplitude of 60 mV. Overall, Sensor 1 shows the smallest variance among the sensors.

To sum up, the SWASV parameters that showed the best Cd^2+^detection of 0.06 ppb LOD, 14.71 μA·ppb^−1^·cm^−2^ sensitivity, and 80 mV FWHM at 10 ppb (Figure 5b) require a step potential of 10 mV, a waveform amplitude of 60 mV, and a frequency of 35 Hz.

### 3.6. Cd^2+^ Sensor Reproducibility in Spiked DI Waters

To validate the sensor’s reproducibility, we ran calibration using nine freshly prepared sensors under the same optimized conditions in spiked DI water (Figure 8). The average sensitivity was 11.19 µA·ppb^−1^·cm^−2^, and the mean LOD was 0.42 ppb. In comparison, the initial three-sensor study conducted under the same optimized conditions yielded an LOD of 0.06 ± 0.02 ppb. This difference does not arise from changes in experimental conditions; rather, the larger nine-sensor dataset captures a broader range of electrode-to-electrode variability associated with independently fabricated sensors. Thus, the 0.06 ± 0.02 ppb value is the LOD from the initial three-sensor evaluation, whereas the mean LOD of 0.42 ppb better represents sensor-to-sensor performance across a larger population of independently prepared electrodes. LODs can vary for independently fabricated sensors because of differences in surface nucleation density, alloy formation efficiency, nanocomposite deposition, and local microstructural variations within the electrode coating. Importantly, the LOD values for all nine sensors were significantly lower than the U.S. EPA drinking-water limit for Cd^2+^ (5 ppb), suggesting that the sensor can achieve sub-ppb detection despite electrode-to-electrode variability. The relative standard deviation (RSD) of 8% indicates good sensor-to-sensor reproducibility and supports the robustness of the sensor fabrication and modification process. Importantly, these results demonstrate that the sensors can provide high sensitivity and reproducible Cd^2+^ detection over independently fabricated electrodes.

### 3.7. Usability of Cd^2+^ Sensors in Real Water Sources

To assess the sensor’s usability for real-world water monitoring, we first investigated its selectivity in the presence of three common interferents, Zn^2+^, As^3+^, and Cu^2+^, by spiking in DI and lake waters (Figure 9a). We added 300 pb of each interferent to the solution containing 30 ppb Cd^2+^, i.e., a 10-fold higher concentration. For DI-spiked samples, without interferents, the highest Cd^2+^ detection current is ~29 µA. This corresponds to a sensitivity of 14.71 ± 0.3 µA·ppb^−1^·cm^−2^ and an LOD of 0.06 ± 0.02 (n = 3). With interferents, the sensors still generated higher detection currents: ~22 µA in the presence of Zn^2+^, ~19 µA in As^3+^, and only ~5.5 µA in Cu^2+^. This translates into a sensitivity of 9.55 ± 0.25, 8.72 ± 18, 2.47 ± 0.09 µA·ppb^−1^·cm^−2^, and 0.23 ± 0.04, 0.46 ± 0.03, 2.21 ± 0.11 ppb of LODs in the presence of Zn^2+^, As^3+,^ and Cu^2+^, respectively. Additionally, we investigated interference from Pb^2+^ and the combined effect of all interferents (i.e., 300 ppb each of Zn^2+^, As^3+^, Cu^2+^, and Pb^2+^) (Figure S6, see Supplementary Information). The Cd^2+^ response was moderately suppressed with a single interferent but most suppressed when all four “mixed” interferents were present simultaneously, emphasizing the importance of understanding complex interference effects for sensor development.

For lake water-spiked samples, in the absence of interferents, we observed voltammograms with distinct, sharp, and undistorted Cd^2+^ anodic peaks (Figure 9b). We noted good linearity (0 to 30 ppb) and a maximum detection current of ~13.34 µA. This corresponds to a sensitivity of 6.94 ± 0.8 µA·ppb^−1^·cm^−2^ and an LOD of 0.61 ± 0.10 (n = 3). With interferents, Zn^2+^ produced the least interference (~12 µA), followed by As^3+^ (~11 µA), and Cu^2+^ with the most interference (~3 µA). This translates to sensitivities of 5.74 ± 0.20, 5.22 ± 0.22, and 1.34 ± 0.06 µA·ppb^−1^·cm^−2^ and corresponding LODs of 2.05 ± 0.04, 2.33 ± 0.33, and 7.86 ± 0.30 ppb in the presence of Zn^2+^, As^3+^, and Cu^2+^, respectively. Figure S6 shows the interference effects from Pb^2+^ and the combined effect of all interferents. One possible solution for real waters that contain high levels of Cu^2+^ is to precipitate (quench) it with ferrocyanide [26] prior to detection, which our lab is currently investigating.

To further validate the electrochemical sensor in the real-water matrix against an EPA-approved device, we directly compared the Cd^2+^ signal recovery from our sensors with a commercial Kemio Palintest platform (Table S4, see Supplementary Information). We spiked East Lake water samples at 1, 5, and 10 ppb Cd^2+^ and analyzed them using both methods. We evaluated three independently prepared electrodes at each concentration and converted the measured stripping currents to Cd^2+^ concentrations using the independently established calibration relationship. The electrochemical sensor measured 0.91 ± 0.27, 4.76 ± 0.67, and 9.01 ± 0.75 ppb for samples spiked at 1, 5, and 10 ppb, respectively, corresponding to recoveries of 91.3%, 95.2%, and 90.1%. The corresponding Palintest measurements were 1.00 ± 0.10, 5.30 ± 0.70, and 9.13 ± 0.99 ppb, with recoveries of 100.0%, 106.0%, and 91.3%, respectively. The electrochemical measurements showed good agreement with Palintest, with relative differences of approximately −9.0%, −10.2%, and −1.3% at 1, 5, and 10 ppb, respectively. Across the three concentration levels, the two methods showed a strong correlation (r = 0.998, R^2^ = 0.996) and an RMSE of approximately 0.32 ppb.

Overall, the Cd^2+^ sensors developed in this work demonstrated excellent usability for detecting Cd^2+^ with high reproducibility and repeatability in natural water matrices, with minimal sample preparation. We are currently performing extensive field testing with other real waters, including well water and treated municipal wastewater. Table 1 compares the Cd^2+^ sensor performance reported in this work with that reported in the literature.

## 4. Conclusions

We have shown that the newly developed ternary nanocomposite coating, when combined with optimal nanocomposite loading and specific SWASV parameters such as deposition potential, frequency, amplitude, and step potential, offers an excellent balance of key performance metrics for Cd^2+^ sensors. This combination is crucial for reliably detecting cadmium in real water sources down to sub-ppb levels, providing high sensitivity (6.94 ± 0.8 μA·ppb^−1^·cm^−2^), selectivity (80 mV FWHM at 10 ppb), reproducibility (8% RSD), and a low LOD (0.61 ± 0.10 ppb) (n = 3). The complementary SEM, EDS, XPS, Raman, and four-probe electrical measurements indicated the importance of electrochemically pretreating the cSPEs, which resulted in I_D_/I_G_ ratios as high as 0.75, a two-fold increase in the electrical conductivity (1.94 × 10^3^ S/m), and a highly uniform and randomly distributed nanocomposite with an average sheet size of 160 nm, Bi particle size of 78 nm, and a surface coverage of ~10.5 ± 0.5%. CV and EIS studies confirmed greater structural disorder, higher defect density, and altered surface functional groups, with a six-fold decrease in *R_ct_* after ECP pretreatment to 109 ± 12 and a 56% decrease in double-layer capacitance impedance after nanocomposite coating. A three-fold increase in the Cd^2+^ detection current (30 μA at 30 ppb) was achieved by applying an optimal nanocomposite loading of 1 mg/mL. By utilizing the new ternary nanocomposite and the optimal SWASV parameters of 10 mV step potential, 60 mV amplitude, and 35 Hz frequency via the fast causal inference algorithm, we demonstrated the best Cd^2+^ detection sensitivity of 14.71 ± 0.3 μA·ppb^−1^·cm^−2^ and an LOD of 0.06 ± 0.02 ppb (n = 3) in spiked DI water. Overall, the Cd^2+^ sensors developed in this work demonstrated their utility for detecting Cd^2+^ with high reproducibility and repeatability in natural water matrices, with minimal sample preparation.

## Figures and Tables

**Figure 1:**
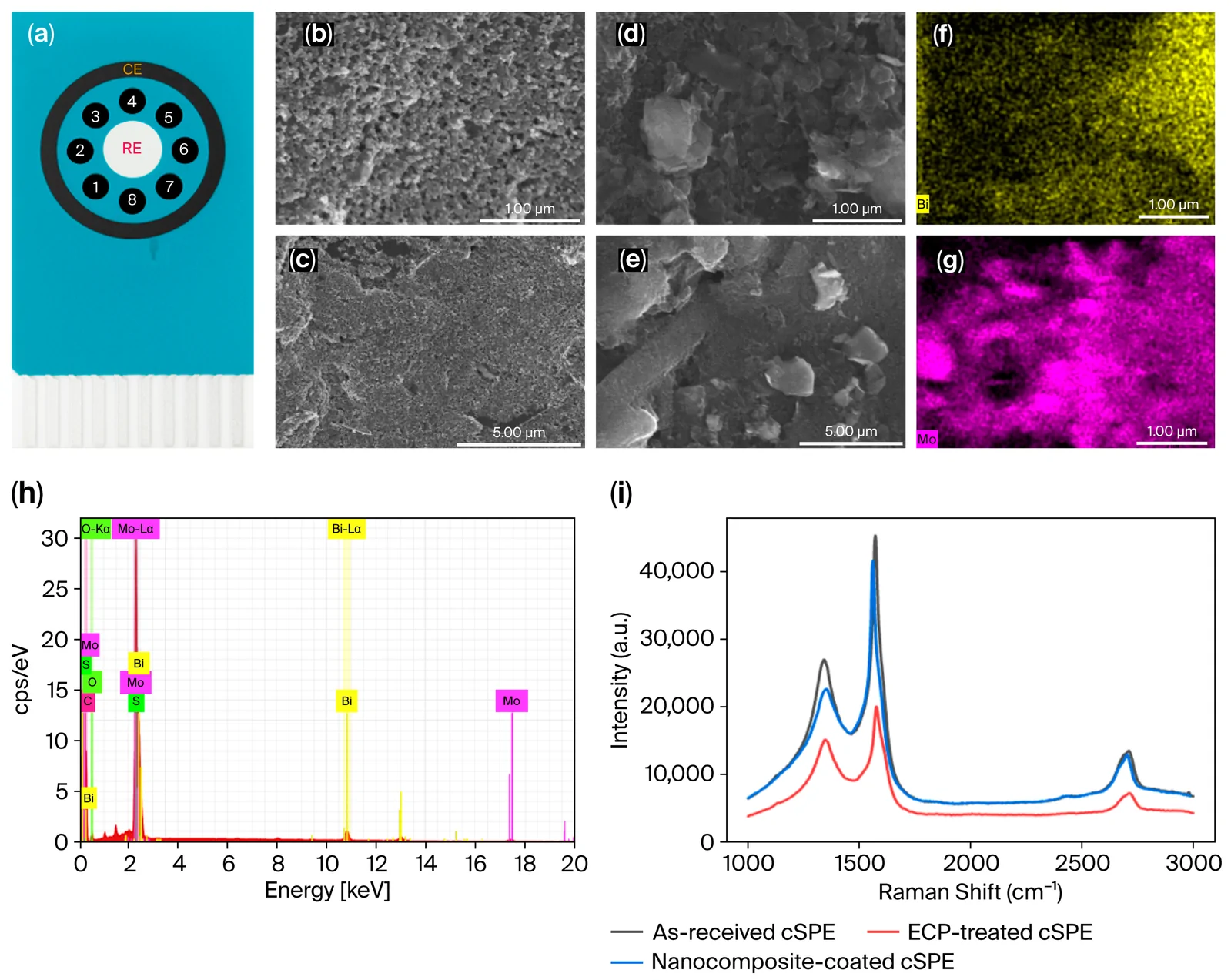
(**a**) cSPE chip with 8 WEs, built–in Ag/AgCl disk reference, and carbon ring counter electrodes. (**b**,**c**) SEM images of unmodified SPCEs; (**d**,**e**) nanocomposite–modified WE; (**f**,**g**) elemental mapping of nanocomposite–modified WE. (**h**) EDS spectral analysis. (**i**) Raman Spectra of cSPE, as-received (black), ECC treated (red), and nanocomposite-modified cSPE. Scale bars for (**b**,**d**) are 5 μm, and scale bars for (**c**,**e**,**f**,**g**) are 1 μm, respectively.

**Figure 2:**
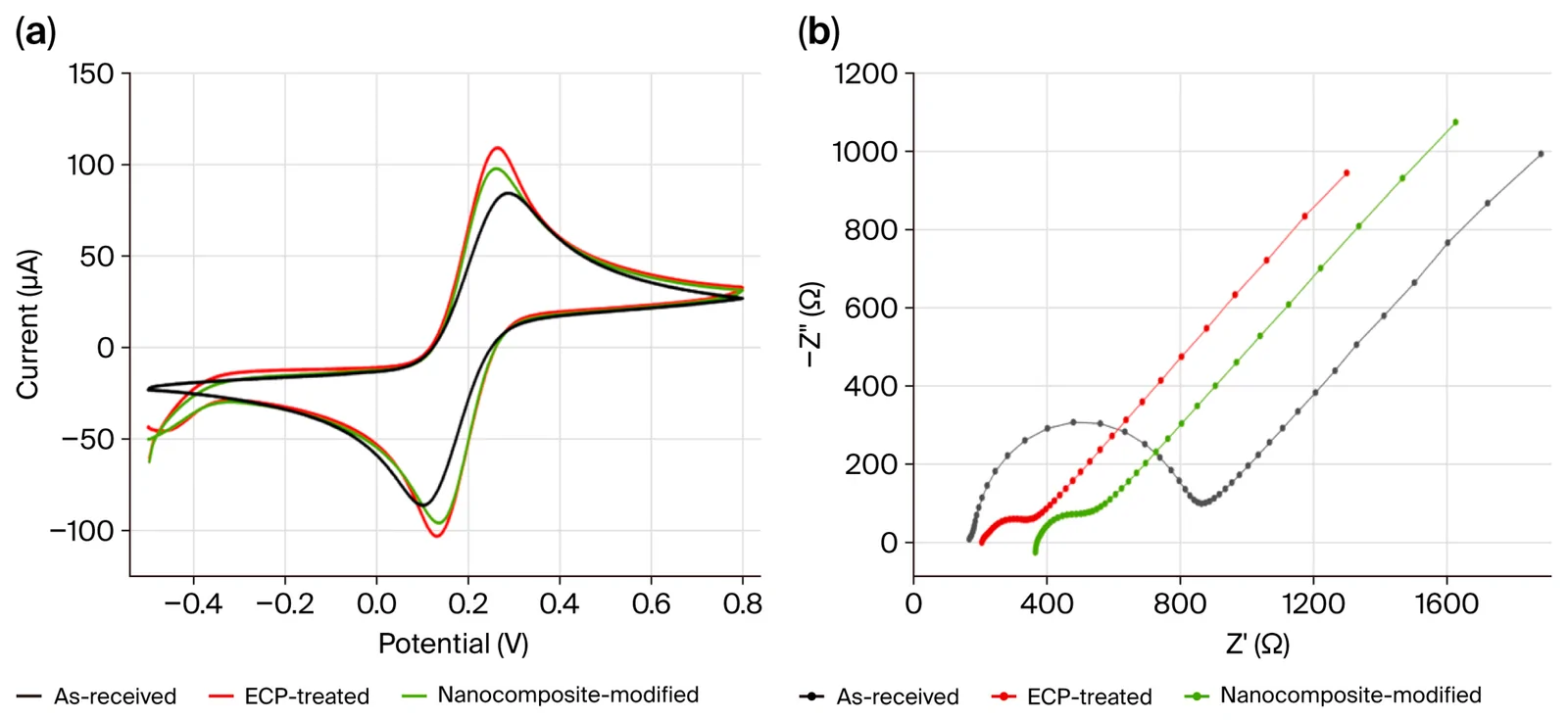
Overlay of (**a**) cyclic voltammograms and (**b**) Nyquist plots recorded for as received cSPE (black), electrochemically polished (ECP) cSPE (red, (BiO)_2_CO_3_–rGO–MoS_2_ coated (green in 5 mM Fe(CN)_6_^3−^/_4_^−^ in 1 M KCl solution at 10 mV amplitude, 0.1 Hz–100 kHz frequency.

**Figure 3:**
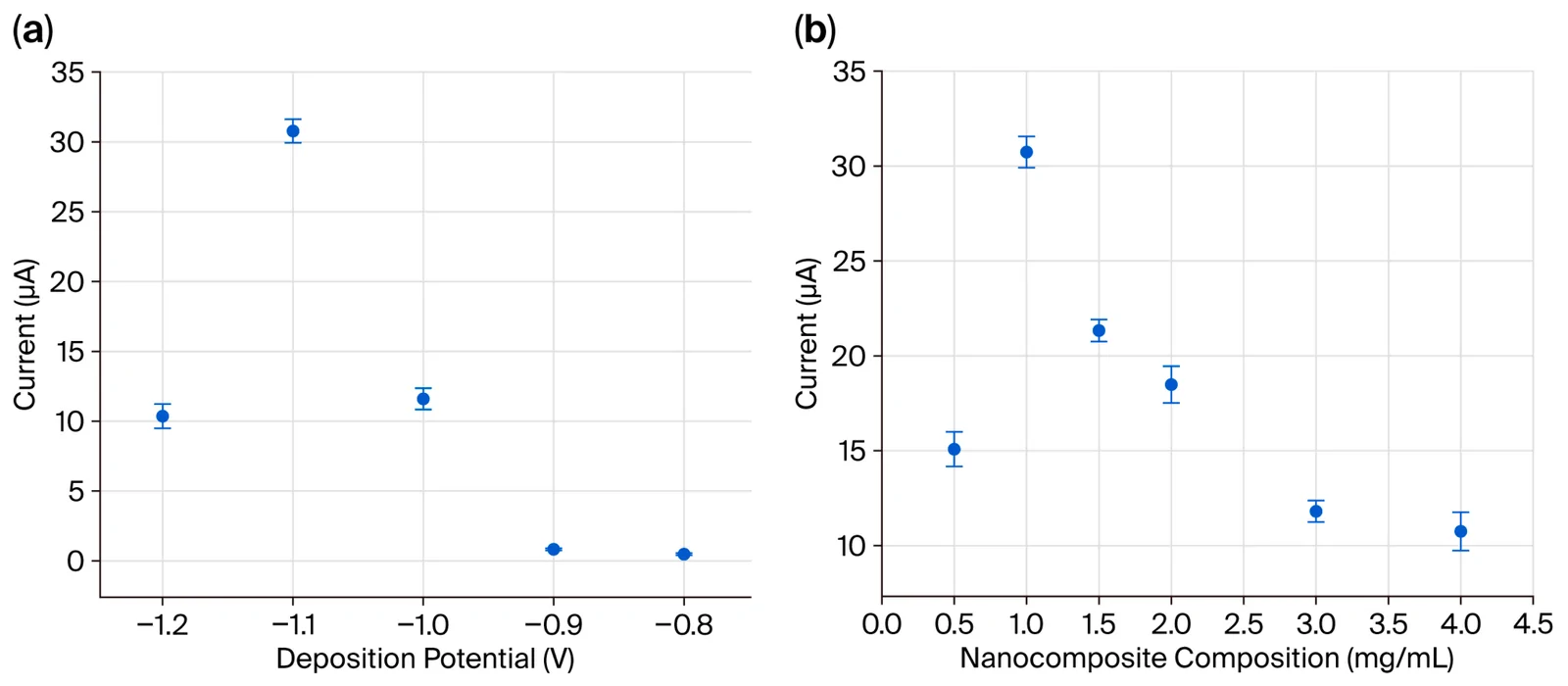
Effect of (**a**) SWASV deposition potential and (**b**) nanocomposite loading. 0.1 M Acetate Buffer Solution made from DI water is used. SWASV frequency 35 Hz, step potential 10 mV, and amplitude 60 mV. Each data point averages three measurements from 3 sensors. The error bars represent ±0.5 μA standard deviation.

**Figure 4:**
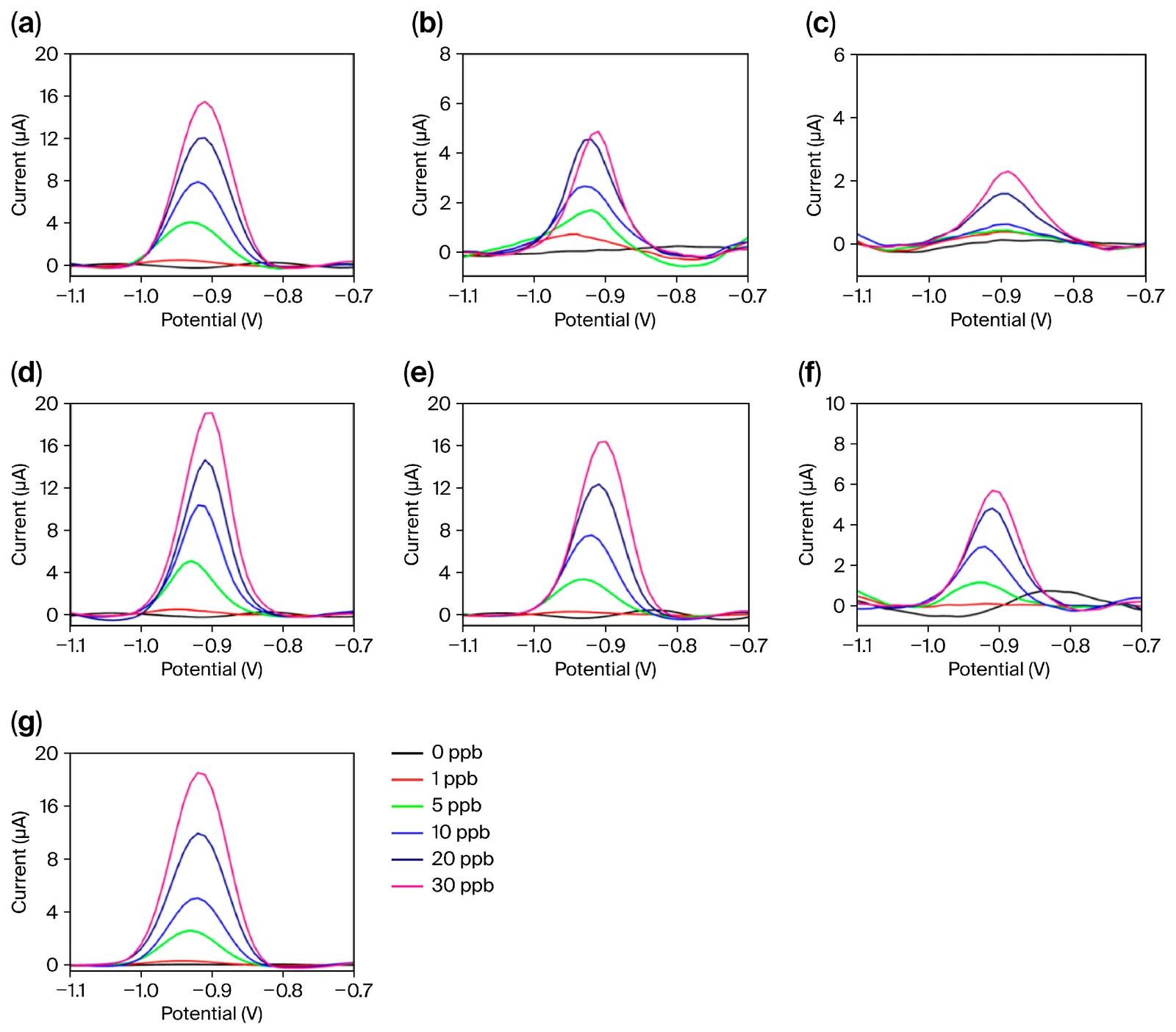
Effect of nanocomposite composition on cadmium sensitivity. SWASV responses of cSPEs modified with (**a**) Bi (**b**) MoS_2_, (**c**) rGO, (**d**) Bi–rGO, (**e**) Bi–MoS_2_, (**f**) rGO–MoS_2_ and (**g**) Bi–rGO–MoS_2_, under identical experimental conditions. Tested in sodium acetate buffer (pH 4.7) at a deposition potential of −1.1 V, deposition time of 200 s, step potential of 10 mV, amplitude of 60 mV, and frequency of 35 Hz.

**Figure 5:**
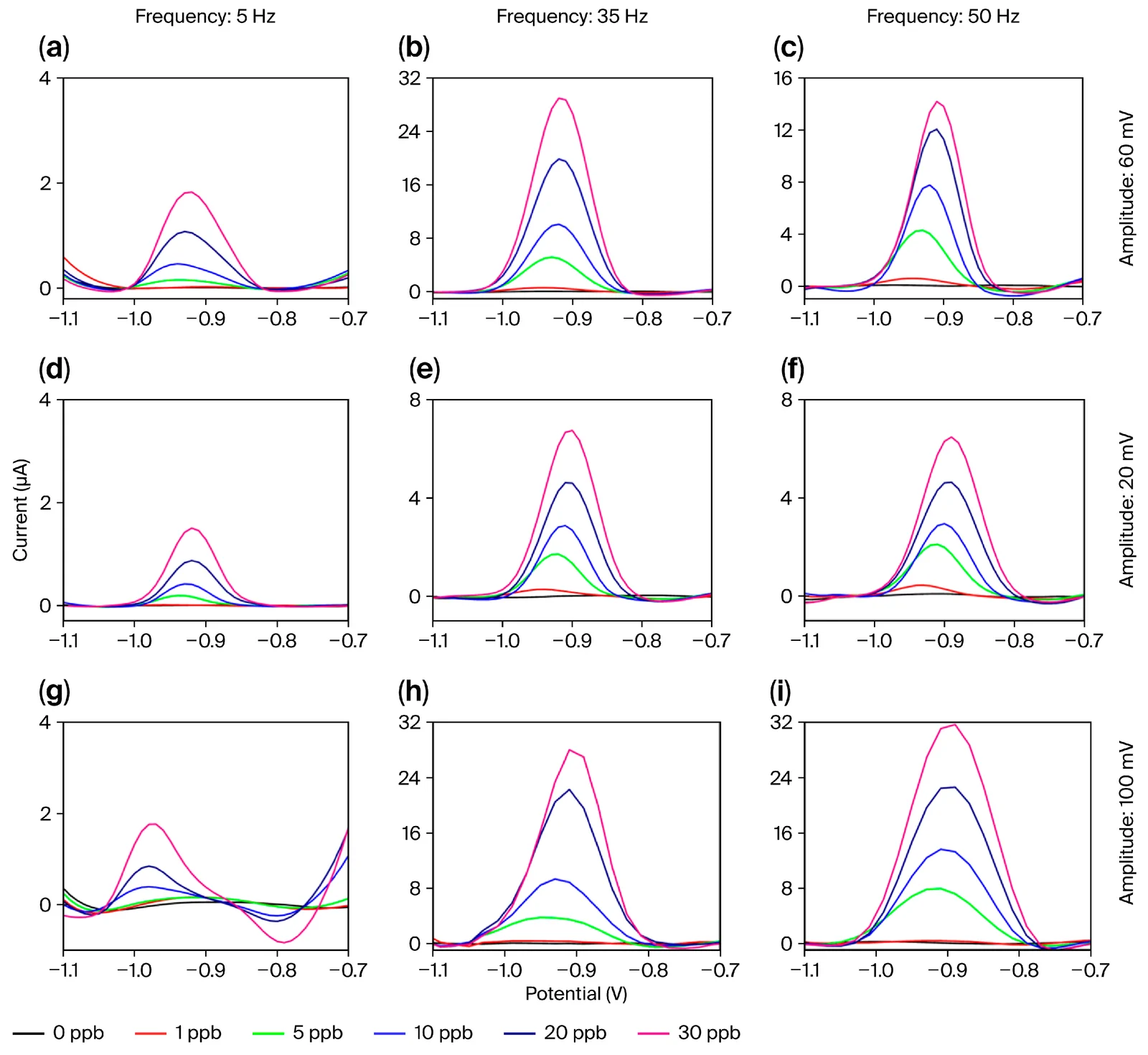
Effect of SWASV parameters on cadmium sensor metrics. (**a**–**c**) Amplitude = 60 mV, and frequencies of 5, 35, and 50 Hz, respectively. (**d**–**f**) Amplitude = 20 mV, and frequencies of 5, 35, and 50 Hz. (**g**–**i**) Amplitude = 100 mV, and frequencies of 5,35 and 50 Hz. For each SWASV condition, the calibration was performed from 0 to 30 ppb in 0.1 M acetate buffer (pH 4.7). The step potential was fixed at 10 mV.

**Figure 6:**
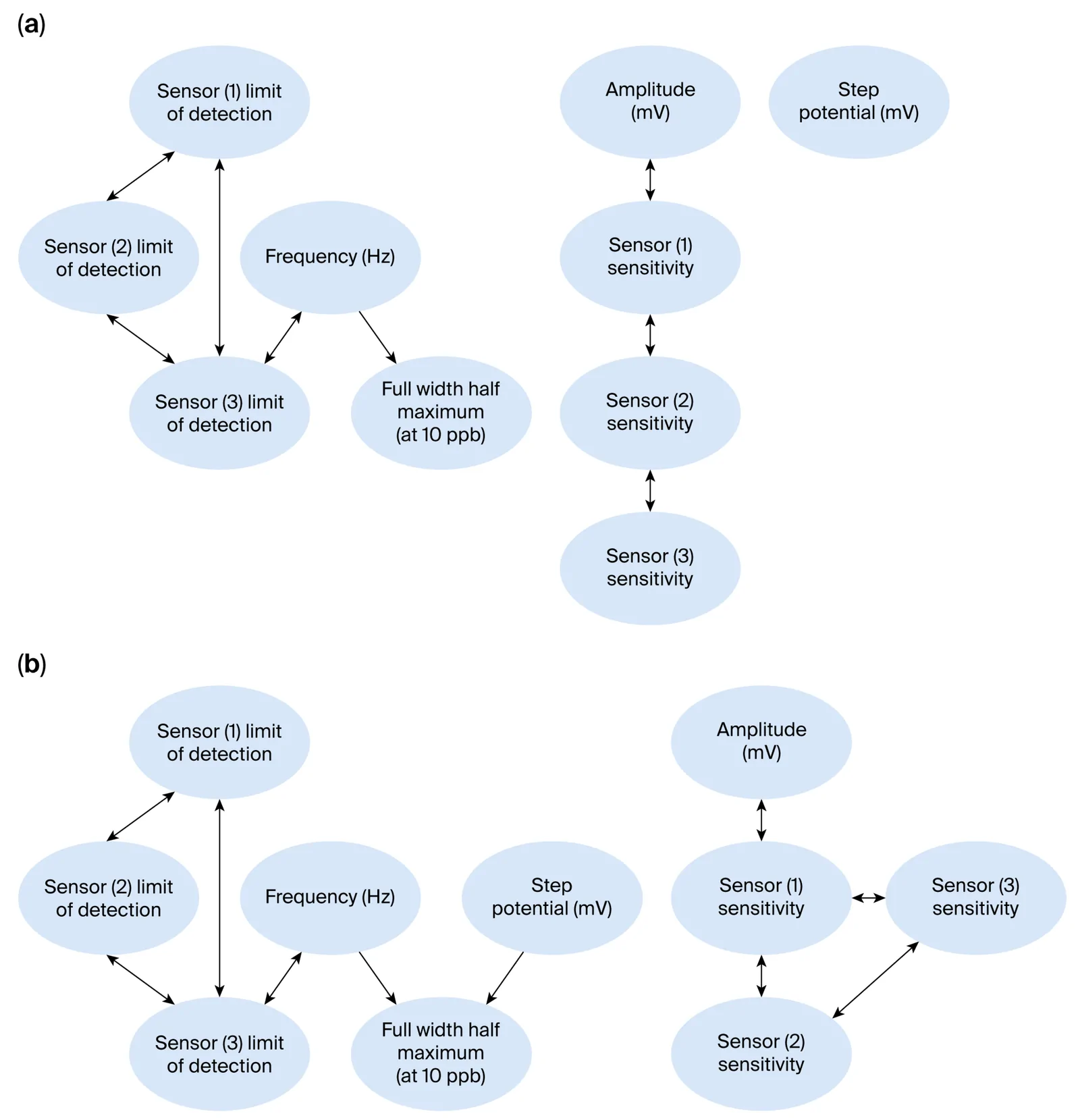
(**a**) FCI algorithm with a Type I error of α = 0.05. The connections between each variable are tested using Fisher’s Z conditional independence test at the α = 0.05 significance level. (**b**) FCI algorithm with a Type I error of α = 0.10. The connections between each variable are tested using Fisher’s Z conditional independence test at the α = 0.10 significance level.

**Figure 7:**
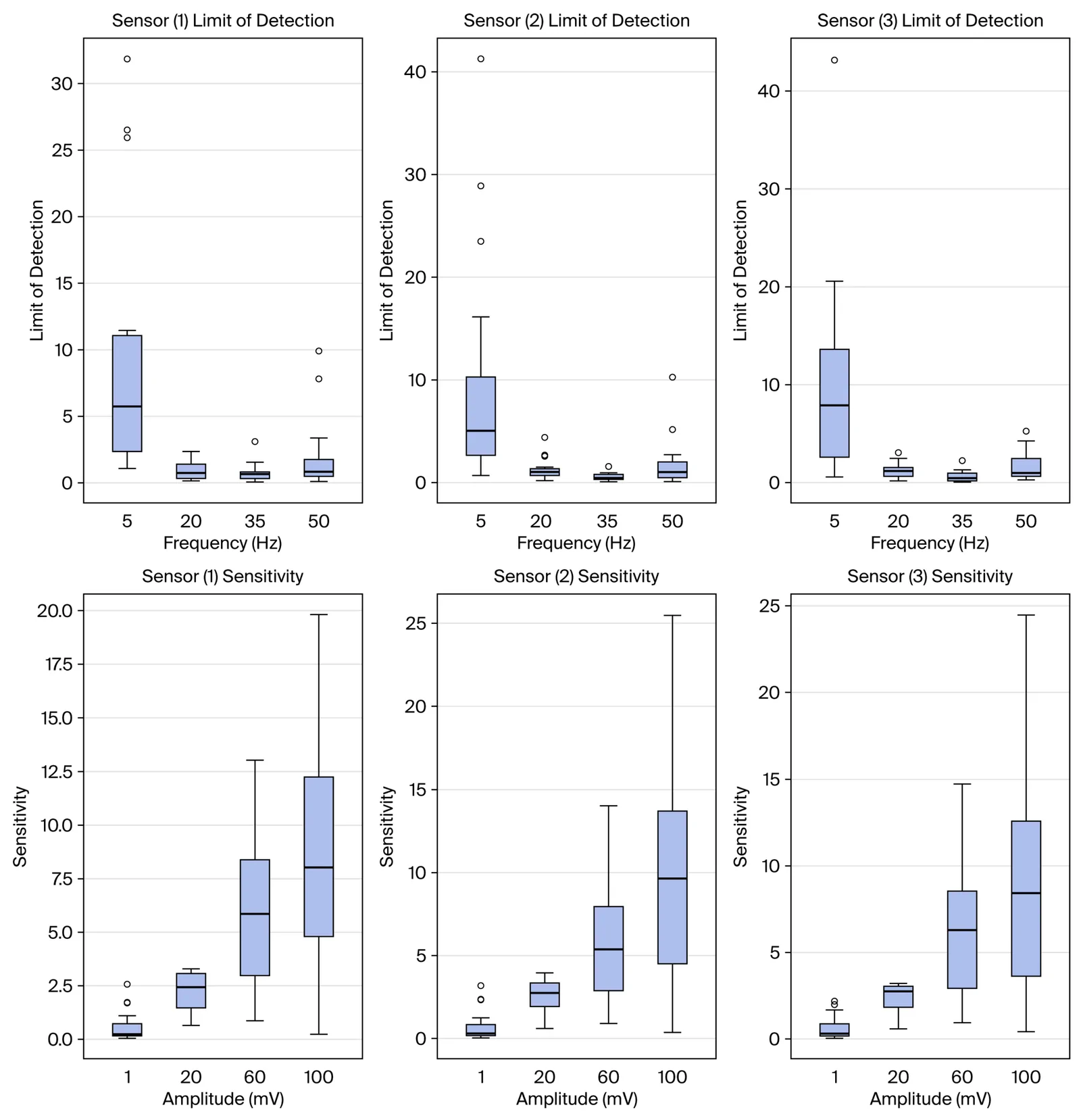
Boxplot of the Cd^2+^ LODs of three sensors grouped by frequency (**top row**) and Cd^2+^ sensitivities of three sensors grouped by amplitude (**bottom row**).

**Figure 8:**
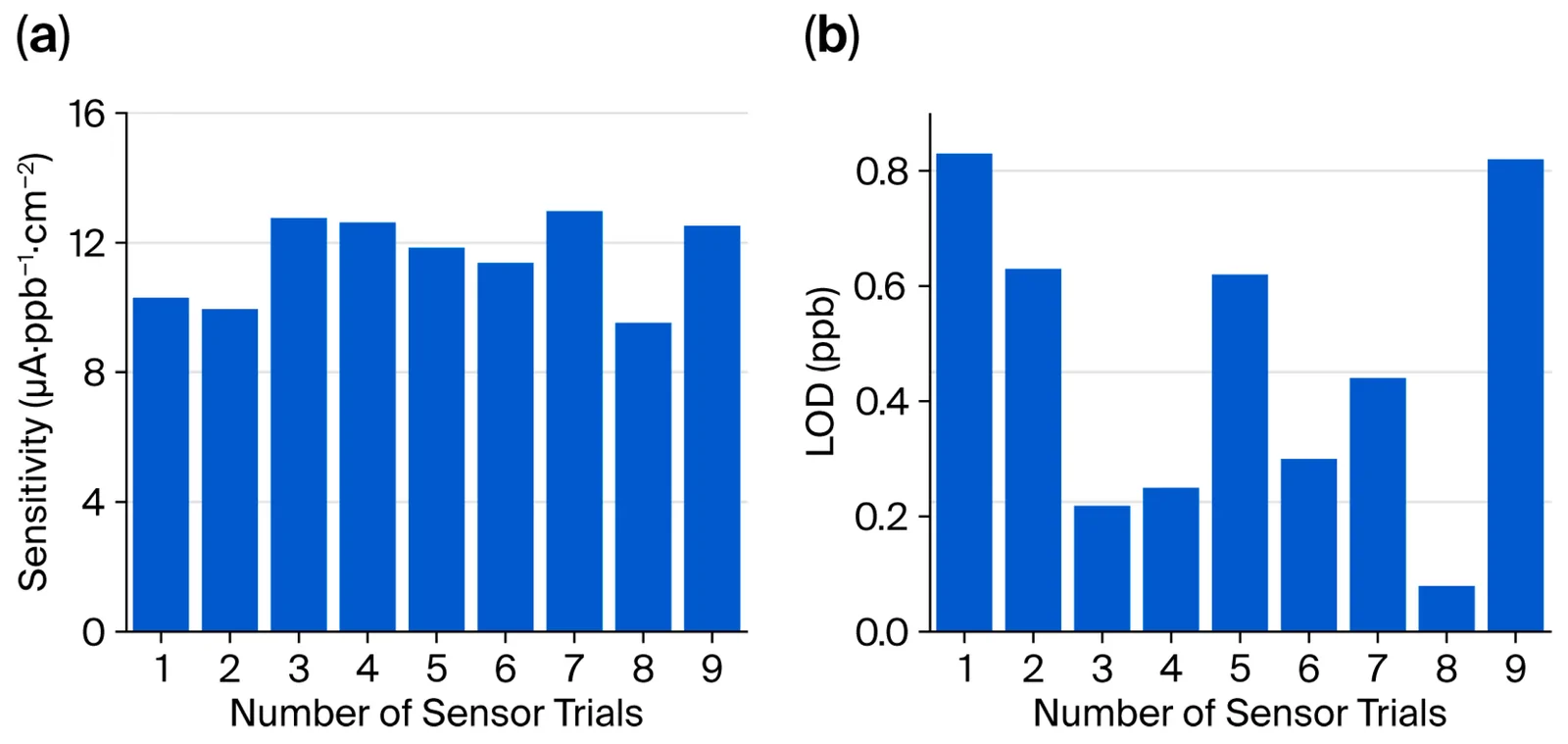
Reproducibility of Cd^2+^ sensors. (**a**) Sensitivity (μA·ppb^−1^·cm^−2^) and (**b**) LOD (ppb) obtained from nine independent sensor trials. Calibration was performed by spiking 0 to 30 ppb Cd^2+^ in DI water with acetate buffer at 4.7 pH.

**Figure 9:**
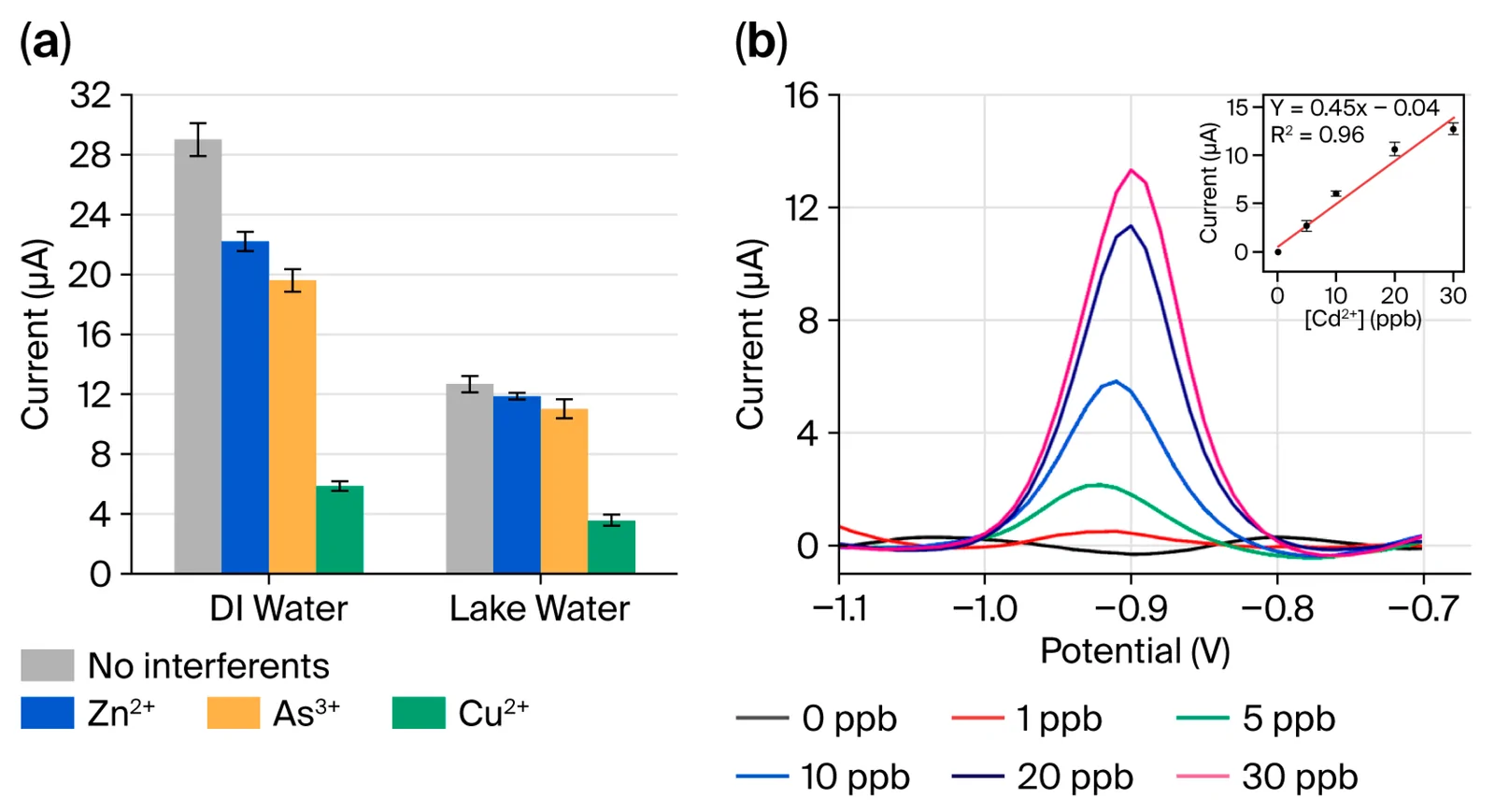
SWASV detection of Cd^2+^ at 30 ppb in 300 ppb of interferent ions (**a**) comparative bar graph illustrating current responses for Zn^2+^, As^3+^, and Cu^2+^ in both water types; (**b**) corresponding voltammetric response in lake water, with no interferents. Error bars represent standard deviations from triplicate measurements (n = 3).

**Table 1:** Comparison of the Cd^2+^ sensor performance achieved in this work with previously reported results.

Electrode Substrate	Linear Range (µg/L)	Analytical Method	LOD (ppb)	Sensitivity (µA/ppb)	References
Bi Oxide SPE	10–150	SWASV	2.5	0.07	[37]
Bi_2_O_3_ SPE	20–100	SWASV	1.5	3.25	[38]
Nano Porous Bi-coated Carbon SPE	10–100	SWASV	1.3	0.137	[39]
Bi/chitosan-coated carbon SPE	1–5	SWASV	0.1	0.522	[40]
(BiO)_2_CO_3_-rGO-Nafion/GCE	1–60	SWASV	0.24	—	[34]
Nafion/IL/GO/GCE	2.4–70	SWASV	0.33	0.0371	[41]
Bi/Sb/GCE	1.0–220	SWASV	0.15	—	[6]
Au-GN-Cys/GCE	0.50–80	SWASV	0.1	—	[42]
Bi_2_O_2_CO_3_-rGO-MoS_2_	0–30	SWASV	0.06	0.1471	This work

## Data Availability

The original contributions presented in this study are included in the article. Further inquiries can be directed to the corresponding author.

## Supplementary Materials

The following supporting information can be downloaded at: https://www.mdpi.com/article/10.3390/s26196112/s1. Figure S1: Images of the cSPE chip and ternary nanocomposite ink; Figure S2: Images of the ternary nanocomposites after the coating with average particle size and surface coverage; Figure S3: XPS analysis of the ternary nanocomposite; Figure S4: Fitted Randles circuit model for as-received, ECP-treated, and nanocomposite-modified cSPE; Table S1: SWASV parameter table for optimization studies; Table S2: Mean and variance of the LOD for each sensor at different SWASV frequencies; Table S3: Mean and variance of the sensitivity for each sensor at different SWASV amplitudes; Figure S5: Scatter plot showing the mean and variance of sensitivity for each electrode across different SWASV amplitude levels; Figure S6: Selectivity studies in the presence of interferents in both DI and lake waters; and Table S4: Comparison of Cd^2+^ concentrations measured using the developed electrochemical sensor and the independent sensor test platform.
