# Supplementary material for: Enhanced Cadmium Detection in Real Water Sources Using a Custom Nanocomposite-Modified Carbon Electrode and Optimized Square Wave Voltammetry
Source: Sensors (Basel). 2026 Sep 26;26(19):6112. doi: 10.3390/s26196112 (PMC13667642; doi:10.3390/s26196112)
Supplement: Supplementary file 1 [file sensors-26-06112-s001.zip › sensors-4545360-supplementary.pdf]

Supporting Information for

Enhanced cadmium detection in real water sources using a custom nanocomposite-modified carbon electrode and optimized square wave voltammetry

Dipen Basnet[1$], Rohit Boddu[1$], Xiyuan Liu[3], Prabhu U. Arumugam[1,2] and Shabnam Siddiqui[2]*

[1]Institute for Micromanufacturing (IfM), Louisiana Tech University, Ruston, LA 71272

[2]Center for Biomedical Engineering and Rehabilitation Science (CBERS), Louisiana Tech University, Ruston, LA 71272

[3]Department of Mathematics and Statistics, Louisiana Tech University, Ruston, LA 71272

[$] First authors contributed equally

[*]Corresponding authors. Email: shabnam@office.latech.edu

Postal address: 911 Hergot Ave, Institute for Micromanufacturing, Louisiana Tech University, Ruston, LA 71272

Supplementary Figure S1

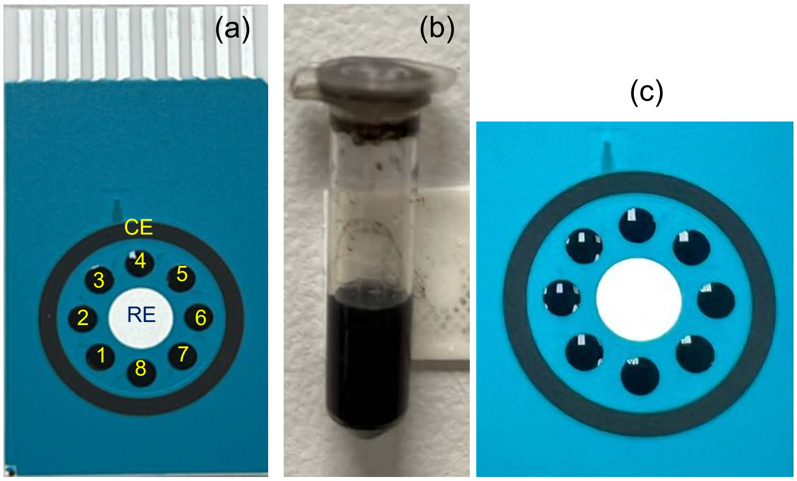

Figure S1. (a) cSPE chip with 8 WEs, built-in Ag/AgCl disk reference electrode, and carbon ring counter electrode. (b) ternary nanocomposite ink and (c) nanocomposite-modified carbon WEs.

Supplementary Figure S2

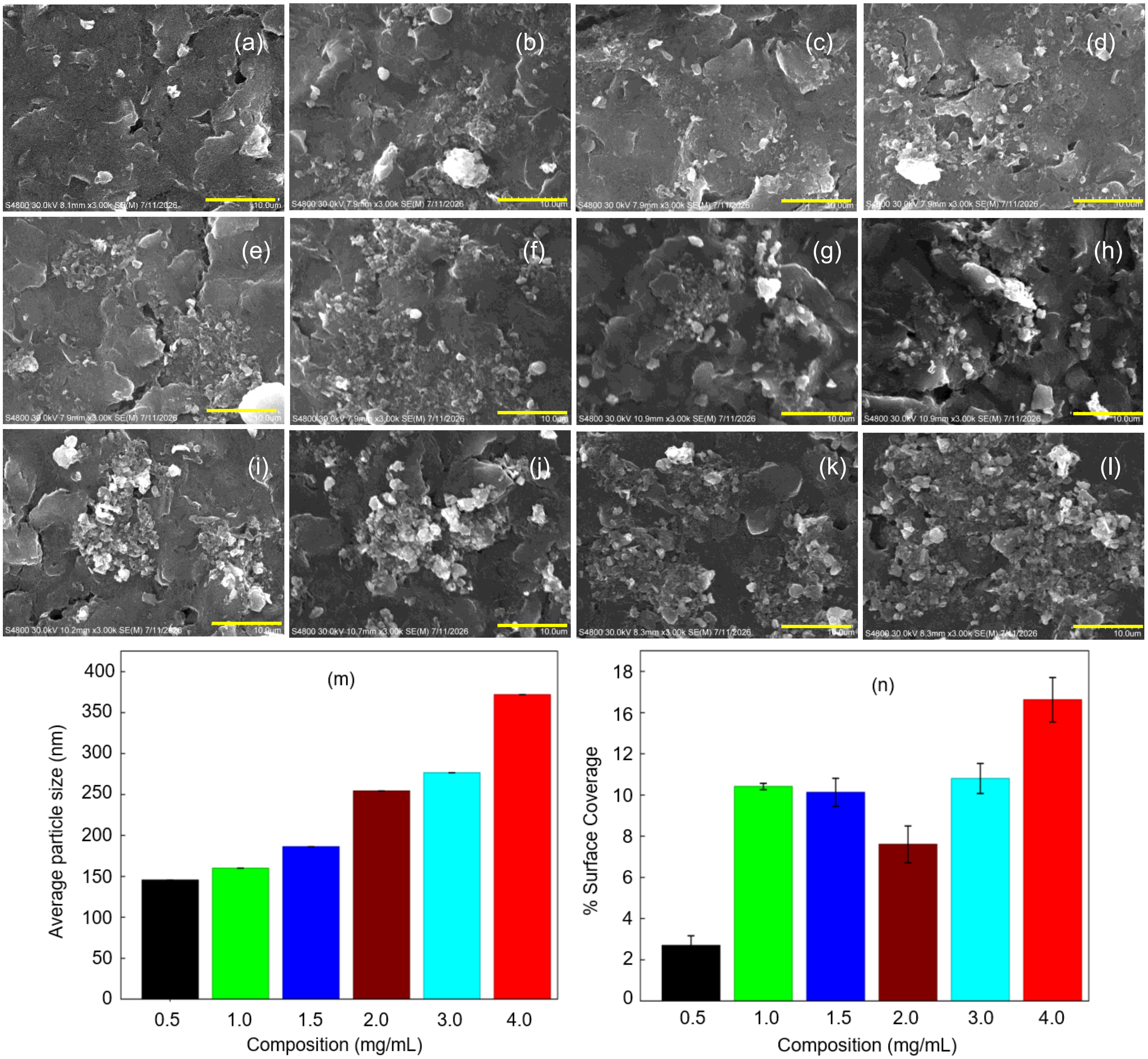

Figure S2. (a–l) SEM micrographs of cSPEs modified with ternary nanocomposites prepared using different loadings: (a, b) 0.5 mg/mL, (c, d) 1.0 mg/mL, (e, f) 1.5 mg/mL, (g, h) 2.0 mg /mL, (i, j) 3 mg/mL, and (k, l) 4.0 mg/mL. Scale bars are 10 μm. (m) Average particle (agglomerate) size measured from SEM images as a function of nanocomposite concentration. (n) Surface coverage (%) of the modified cSPE determined from SEM image analysis. Error bars represent the standard deviation obtained from measurements performed on replicate SEM images (n=5).

Supplementary Figure S3

Figure S3. XPS analysis of the ternary ($Bi_2O_2CO_3$)-rGO-MoS_2 nanocomposite custom synthesized in the lab.

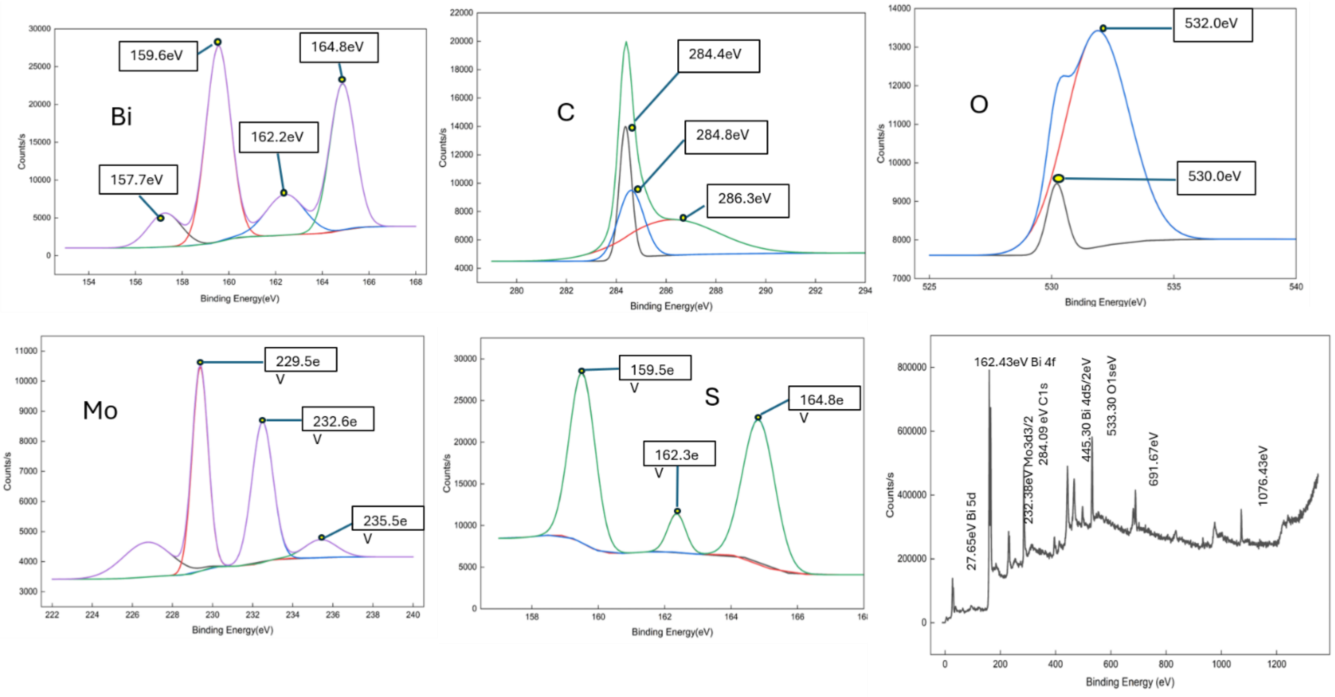

Region	Binding Energy (eV)	Results
**Bi 4f**	157.7	Reduced Bi species
**Bi 4f**	159.6	Bi^{3+} $4f_{7/2}$ (Bi-O)
**Bi 4f / S 2p**	162.2	S2- $2p_{3/2}$ / overlap region
**Bi 4f**	164.8	Bi^{3+} $4f_{5/2}$ (Bi-O)
**C 1s**	284.4	C=C (sp^2 carbon, rGO)
**C 1s**	284.8	C-C / adventitious carbon
**C 1s**	286.3	C-O / C-O-C
**O 1s**	530.0	Metal-O (Bi-O and/or Mo-O)
**O 1s**	532.0	C-O / OH / carbonate-related oxygen
**Mo 3d**	~226.7	S 2s
**Mo 3d**	229.5	Mo^{4+} $3d_{5/2}$ (MoS2)
**Mo 3d**	232.6	Mo^{4+} $3d_{3/2}$ (MoS2)
**Mo 3d**	235.5	Mo^{6+} $3d_{3/2}$ / oxidized Mo
**S 2p / Bi 4f**	159.5	Bi^{3+} $4f_{7/2}$, not sulfur
**S 2p**	162.3	S2- $2p_{3/2}$ (MoS2)
**S 2p**	~163.5	S2- $2p_{1/2}$ (MoS2)
**S 2p / Bi 4f**	164.8	Bi^{3+} $4f_{5/2}$, not sulfur

Supplementary Figure S4

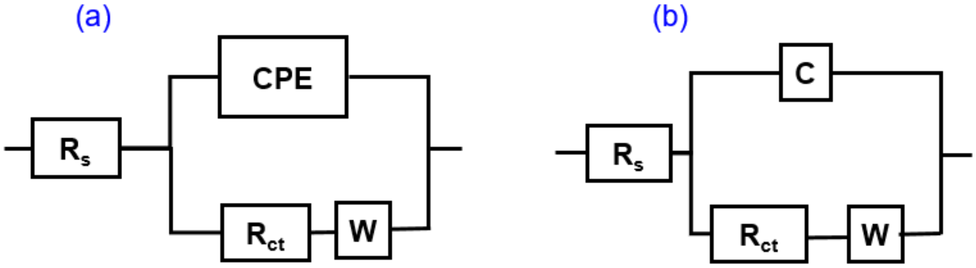

Figure S4. Fitted Randles circuit model for (a) as-received cSPE and (b) ECP-treated and nanocomposite-modified cSPE.

Supplementary Table S1

ΔE_p (mV)	ΔE_s (mV)	→					
0		5	10	20	30	40	50
20		5	10	NE	NE	NE	NE
60	5	10	20	30	40	NE	NE
100	5	10	20	30	50	70	NE

We started with a pulse frequency of 5 Hz. The scan time for ΔE_s = 5 mV at this frequency would be 34 s. However, at 50 Hz, it would be 3.4 s. So, scan time would be shorter with higher frequencies. In the table above, NE means no experiment for those conditions. Since such conditions do not satisfy $\Delta E_s \ll \Delta E_p$. A total of 19 experiments for each frequency. The total number of experiments is 76 (19 x 4) at the following frequencies: 5, 20, 35, and 50 Hz.

Supplementary Table S2

Mean and variance of the LOD for each sensor at different frequencies.

Sensor 1			Sensor 2			Sensor 3		
Mean	Var	Freq	Mean	Var	Freq	Mean	Var	Freq
8.97	85.46	5	9.43	118.34	5	10.41	103.98	5
0.94	0.44	20	1.23	1.03	20	1.22	0.59	20
0.75	0.47	35	0.55	0.18	35	0.59	0.31	35
1.85	7.01	50	1.75	5.67	50	1.62	2.03	50

Supplementary Table S3

Mean and variance of the sensitivity for each sensor at different amplitudes.

Sensor 1			Sensor 2			Sensor 3		
Mean	Var	Amp	Mean	Var	Amp	Mean	Var	Amp
0.542	0.412	1	0.68	0.68	1	0.6	0.38	1
2.185	1.125	20	2.48	1.51	20	2.29	1.17	20
6.192	15.02	60	5.95	16.23	60	6.45	19.7	60
8.476	33.96	100	9.37	48.37	100	8.71	45.45	100

Supplementary Figure S5

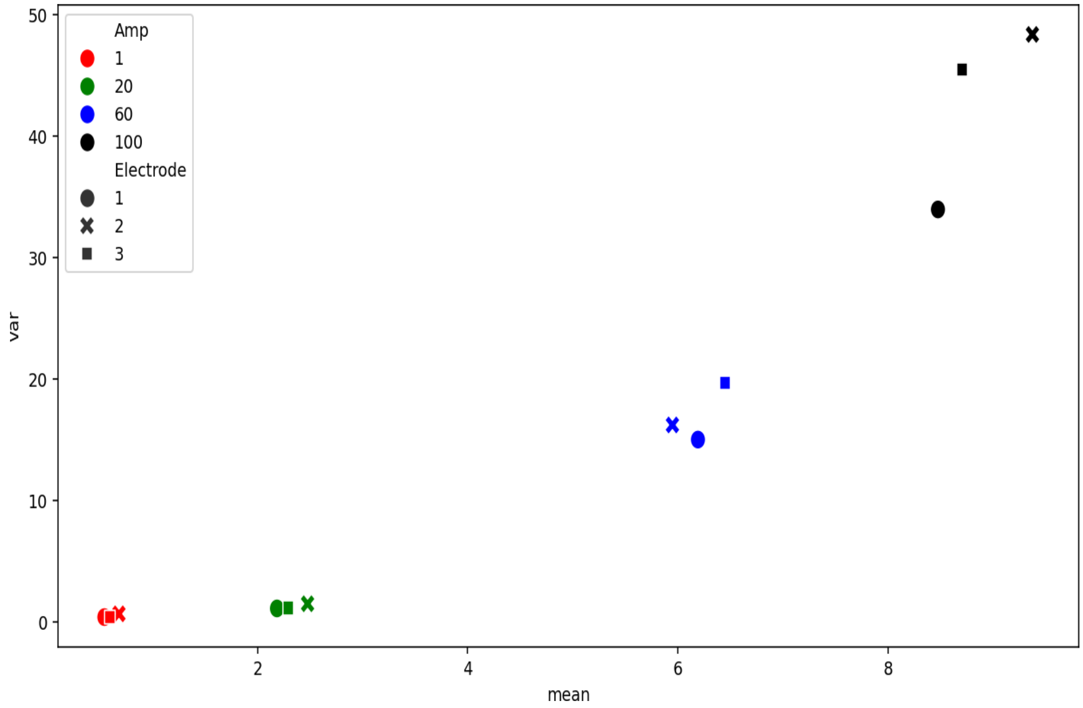

Figure S5. Scatter plot showing the mean and variance of sensitivity for each electrode across different amplitude levels.

Supplementary Figure S6

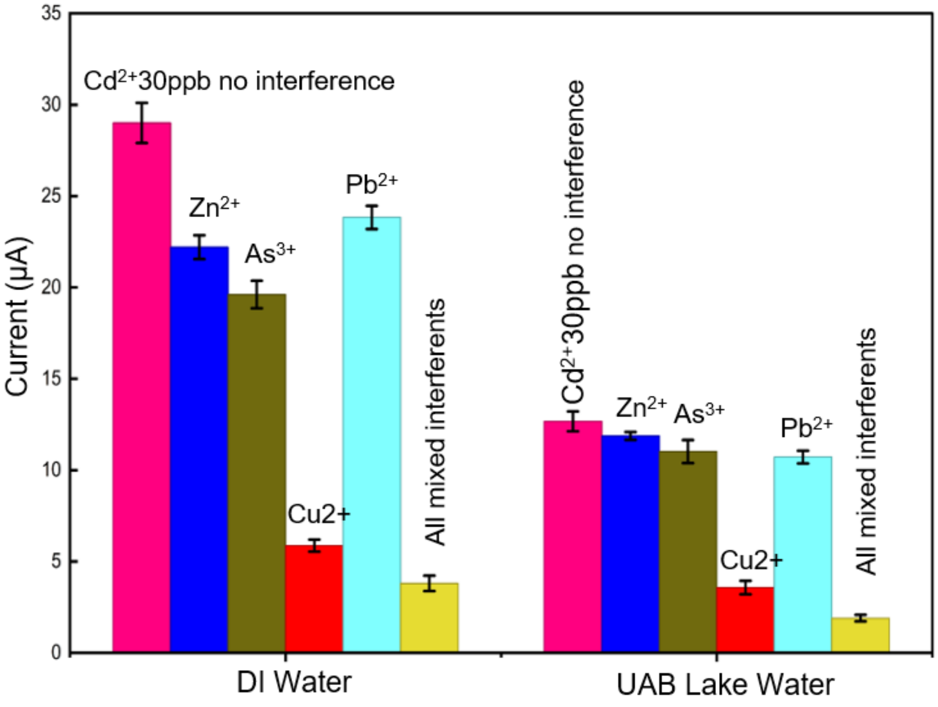

Figure S6. Selectivity studies. Comparative bar graph illustrating of Cd^{2+} current responses (30 ppb) for 300 ppb each of Zn^{2+}, As^{3+}, Cu^{2+} Pb^{2+} and all interferents combined at 300 ppb each "all mixed interferents" in both water types. Error bars represent standard deviations from triplicate measurements (n=3).

Supplementary Table S4

Table S4: Comparison of Cd^{2+} concentrations measured using the developed electrochemical sensor and the independent Kemio Palintest platform in spiked East Lake water. Values represent mean ± standard deviation from three measurements. Recovery was calculated relative to the nominal spiked Cd^{2+} concentration.

Spiked Cd^{2+} (ppb)	Palintest measured Cd^{2+} (ppb)	Palintest recovery (%)	Sensor measured Cd^{2+} (ppb)	Sensor RSD (%)	Sensor recovery (%)	Difference vs Palintest (%)
1	1.00 ± 0.10	100.0	0.91 ± 0.27	29.1	91.3	−8.7
5	5.30 ± 0.70	106.0	4.76 ± 0.67	14.0	95.2	−10.2
10	9.13 ± 0.99	91.3	9.01 ± 0.75	8.3	90.1	−1.3
